# Enhanced Activation of Canonical Wnt Signaling Confers Mesoderm-Derived Parietal Bone with Similar Osteogenic and Skeletal Healing Capacity to Neural Crest-Derived Frontal Bone

**DOI:** 10.1371/journal.pone.0138059

**Published:** 2015-10-02

**Authors:** Shuli Li, Natalina Quarto, Kshemendra Senarath-Yapa, Nathaniel Grey, Xue Bai, Michael T. Longaker

**Affiliations:** 1 Hagey Laboratory for Pediatric Regenerative Medicine, Department of Surgery, Stanford University, School of Medicine, Stanford, CA, United States of America; 2 Dipartimento di Scienze Biomediche Avanzate, Universita’ degli Studi di Napoli Federico II, Napoli, Italy; University of Texas Southwestern Medical Center, UNITED STATES

## Abstract

Bone formation and skeletal repair are dynamic processes involving a fine-tuned balance between osteoblast proliferation and differentiation orchestrated by multiple signaling pathways. Canonical Wnt (cWnt) signaling is known to playing a key role in these processes. In the current study, using a transgenic mouse model with targeted disruption of *axin2*, a negative regulator of cWnt signaling, we investigated the impact of enhanced activation of cWnt signaling on the osteogenic capacity and skeletal repair. Specifically, we looked at two calvarial bones of different embryonic tissue origin: the neural crest-derived frontal bone and the mesoderm-derived parietal bone, and we investigated the proliferation and apoptotic activity of frontal and parietal bones and derived osteoblasts. We found dramatic differences in cell proliferation and apoptotic activity between *Axin2* ^*-/-*^ and wild type calvarial bones, with *Axin2* ^*-/-*^ showing increased proliferative activity and reduced levels of apoptosis. Furthermore, we compared osteoblast differentiation and bone regeneration in *Axin2* ^*-/-*^ and wild type neural crest-derived frontal and mesoderm-derived parietal bones, respectively. Our results demonstrate a significant increase either in osteoblast differentiation or bone regeneration in *Axin2* ^*-/-*^ mice as compared to wild type, with *Axin2* ^*-/-*^ parietal bone and derived osteoblasts displaying a “neural crest-derived frontal bone-like” profile, which is typically characterized by higher osteogenic capacity and skeletal repair than parietal bone. Taken together, our results strongly suggest that enhanced activation of cWnt signaling increases the skeletal potential of a calvarial bone of mesoderm origin, such as the parietial bone to a degree similar to that of a neural crest origin bone, like the frontal bone. Thus, providing further evidence for the central role played by the cWnt signaling in osteogenesis and skeletal-bone regeneration.

## Introduction

Large defects in cranial or facial bones represent a major challenge in reconstructive surgery [1, 2]. Children less than 2 years of age are capable of healing large calvarial defects, whereas adults lack this endogenous ability[3] Calvarial healing and underlying mechanism(s) have been widely studied both *in vivo* and *in vitro* [4–9]. Several signaling pathways are known to play important roles in calvarial osteogenesis, including TGF-β, BMP, FGF, and Wnt signaling pathways[6, 8–16] Using a calvarial frontal and parietal bone mouse model we have previously demonstrated that the neural-crest derived frontal bone and osteoblast cells (FOb) are endowed with greater osteogenic potential and tissue repair than the mesoderm-derived bone and osteoblasts (POb) [4, 6, 8, 9]. Our studies unveiled integration of multiple and converging osteogenic signaling pathways which are differentially activated between frontal and parietal bones to contribute to the skeletal differences observed between these two calvarial bones [17]. We found both, *in vitro* and in *vivo*, enhanced activation of endogenous FGF, BMP and cWnt signaling pathways in the neural crest-derived frontal bone compared to the mesoderm-derived parietal bone. In contrast, the latter bone displayed only increased activation of endogenous TGF-β signaling [9, 17].

The cWnt signaling pathway is an important regulator of cellular differentiation in a variety of cell types, including osteoblasts [16, 18]. It plays a widespread role in skeletogenesis, spanning from embryonic skeletal patterning through fetal skeletal development and bone remodeling. Wnt ligands are a family of secreted glycoproteins which bind to seven-span trans-membrane receptors called Frizzleds (Fzds) and single span co-receptor proteins LRP–5/6[15]. Binding of Wnt ligands to the receptor complex, triggers activation of the intracellular protein Dishevelled (Dvl) [15]. Activation of Dvl leads to the inhibition of glycogen synthase kinase 3β (GSK–3β) preventing β-catenin degradation by the protein complexes consisting of GSK–3β, axin, and adenomatous polyposis coli (APC)[15].

Our previous investigation identified both *in vitro* and *in vivo* enhanced endogenous cWnt signaling in frontal bone compared to parietal bone, and demonstrated that constitutive activation of cWnt signaling in paraxial mesoderm-derived parietal bone osteoblasts mimics the osteogenic potential of neural crest-derived frontal bone osteoblasts[6]. In contrast, knockdown of canonical Wnt signaling dramatically impaired the greater osteogenic potential of neural crest-derived frontal osteoblasts. Based on these previous observations, we next sought to investigate *in vivo* the role on cWnt signaling on frontal and parietal bones repair. To this end, we have employed a calvarial defect healing model in transgenic mouse with targeted disruption of Axin2 [19], an inhibitor of cWnt signaling. The current work also investigated the effect of enhanced activation of cWnt signaling on proliferation and apoptosis in frontal and parietal bones and derived osteoblasts. The results obtained from the study demonstrate that enhanced activation of cWnt confers to mesoderm-derived bone and osteoblasts an osteo/skeletal potential similar to that of wild type frontal bone. Furthermore, our data indicate that the higher osteo/skeletal potential gained by *Axin2* ^*-/-*^ mesoderm-derived bone and POb was paralleled by increased proliferation and decreased apoptosis.

## Materials and Methods

### Animals

All experiments with animals were performed following Stanford University Animal Care and Use Committee guidelines. All research on animals has been approved by Stanford APLAC, protocol #9999, following approved guidelines by the Stanford University's Institutional Review Board. CD–1 wild type mice were purchased from Charles River Laboratories Inc. *Axin2* ^(LacZ/LacZ^) homozygotic mice, were genotyped as previously described [19]. Herein, these mice are referred as to *Axin2* ^*-/-*^. Animals were housed in light- and temperature-controlled rooms and were given food and water *ad libitum*.

### Tissue harvesting and primary cell cultures

Neural crest-derived frontal osteoblasts (FOb) and mesoderm-derived parietal osteoblasts (POb) were derived from *Axin2* ^*-/-*^ and wild type skulls harvested from mice at postnatal day 21 (pN21), as previously described [6, 8]. Prior enzymatic digestion, the pericranium and dura mater were stripped off from the skull and the cranial suture were also removed. Frontal and parietal bones were minced separately into small chips less than 1 mm. Bone chips were then digested with 0.2% Dispase II and 0.1% Collagenase A (Roche Diagnostics, Indianapolis, IN, USA) in serum-free medium. The digestion was carried out 6 times, each 10 minutes. The first 2 digestions were discarded. The later four digestions were pooled together. All digestions were neutralized with an equal volume of α-MEM supplemented with 10% fetal calf serum (FCS), (Gemini Bioproducts, Woodland, CA), 100 IU/ml penicillin and streptomycin (GIBCO, Invitrogen Corporation, Carlsbad, CA), pelleted and resuspended in growth medium. Both FOb and POb cells were plated in 100 mm tissue culture dishes (Corning Incorporated, New York, NY) and incubated at 37°C with continuous supplement of 5% CO_2._ The medium was changed every other day. Only passage 0 and 1 cells were used for all experiments. BrdU assay was performed as previously described [9]. Samples were run in triplicates and the assay was repeated twice.

### Osteogenic differentiation assay

FOb or POb derived *Axin2* ^*-/-*^ and wild type mice were plated in 6-well-plate (1–2 x 10^5^ cells / well). Upon sub-confluence, cells were incubated with the osteogenic differentiation medium (ODM), made of α-MEM supplemented with 10 μM glycerol β-phosphate, 0.25 μM ascorbic acid, (Sigma Aldrich, St. Louis, MO), 10% FCS, and 1% penicillin/streptomycin. The medium was changed every other day. Mineralization of the extracellular matrix was assessed by alizarin red staining at day 18 of the differentiation followed by its quantification as previously described[6, 9]. The assay was run in triplicates and repeated four times. All morphological observations and analysis were conducted by using Leica DMIL microscope and Leica Microsystems digital imaging software (Leica Microsystems Wetzlar, Germany).

### RNA isolation, reverse-transcriptase polymerase chain reaction and PCR analysis

Total RNA was isolated from cells by the TRIzol method (Invitrogen, Carlsbad, CA. Reverse transcription (RT), PCR analysis and primers sequences were described previously [20–22]. Densitometry analysis of electrophoretic bands was performed using the Image J software program, (NIH, Bethesda, MA). The density of each band was normalized to the housekeeping gene *Gapdh* and presented as percentage increase or decrease. The results are the mean ±SD of three independent experiments. Each samples was run in triplicates.

### Measurement of Caspase 3 activity

Caspase 3 fluorometric protease assay was performed using a Caspase–3 Apoptosis Detection Kit (sc–4263 AK, Santa Cruz Biotechnology) according to the manufacturer’s instructions. Cell lysates (10^6^ cell/0.5ml) were collected at different time points, 40 µl of cell lysates (in triplicate) were incubated with 200 μl of reaction buffer, 5 μl of EDVD-AFC substrate and DTT (final concentration of 10 mM) at room temperature for 1 hour. The analysis was conducted using a fluorescent microplate reader (SpectraMAX Gemini XS, Molecular Devices Corporation, CA, USA) at excitation/emission wavelength of 400/505 nm. Levels of emission of FOb and POb were compared.

### Immunoblotting analysis

FOb and POb cells were collected at different time points of the osteogenic differentiation assay and lysated with cold RIPA buffer (50mmol/L of HEPES, pH 7.5, 150mmol/L of NaCl, 1mmol/L of EDTA, 10% glycerol, 1% Triton-X–100, 25mM sodium fluoride) containing 1 mM sodium orthovanadate and proteases inhibitor cocktail (Sigma-Aldrich, St. Louis, MO). Cell lysates (40 μg) were electrophoresed on 12% Tris-HCl sodium dodecyl sulfate (SDS)-PAGE gels (Precast Nupage gels, Gibco Life Technologies and Invitrogen Corporation, Carlsbad, CA) and transferred onto Immobilon-P membrane (Millipore Corporation, Bedford, MA). Immunoblotting analysis was performed using as primary rabbit antibody a rabbit anti-Caspase 3 antibody (sc–7148), (dilution 1:200, Santa Cruz Biotechnology, Santa Cruz, CA). As secondary antibody was used a horseradish peroxidase-conjugated anti-rabbit antibody (dilution 1:2000, Cell Signaling Danvers, MA). Immunoblotted proteins were visualized by enhanced chemiluminescence (Amersham Biosciences, Buckinghamshire, UK). To control for equal loading and transfer of the samples the membranes were stripped and reprobed with anti-α-tubulin antibody (ab 8227), (dilution 1:1000, Abcam, Camdridge, MA). Densitometry analysis of electrophoretic bands was performed using the ImageJ software program (NIH, Bethesda, MA). The density of each Caspase 3 band was normalized to the loading control (α-tubulin) and presented as percentage increase. The results are the mean ±SD of three independent experiments.

### Indirect immunofluorescent staining

FOb and POb cells were seeded at low density on circle glass coverslips (12 mm) in triplicate and placed in 6-well plates with growth medium (α-MEM, 10% FBS, 1% penicillin and streptomycin). After overnight culture, medium was changed and replaced with ODM. A set of control cells were maintained in growth medium. For all cells the medium was changed every other day. At day 3, 6, and 12, cells were washed twice with phosphate buffered saline (PBS) and fixed with 50% acetone–50% methanol for 20 minutes at 4°C, followed by washing with PBS–0.1%Triton–100 twice. Then, cells were incubated in a blocking solution of 1% horse serum in PBS–0.05% Tween–20 for 1 hour at room temperature followed by incubation with the primary antibody anti-Caspase 3 (sc–7148), (dilution 1:50, Santa Cruz Biotechnology, Santa Cruz, CA) or anti-active β-catenin (anti-ABC), clone 8E7 (1:400; Millipore, Tamecula, CA), overnight at 4°C. Sub sequentially, cells were washed three times with PBS/0.1% Tween–20 and incubated in the blocking solution for 1 hour at room temperature followed by incubation with donkey fluorescein-conjugated anti-rabbit IgG secondary antibody Alexa-fluor 488, (dilution 1:2000, Molecular Probes, Invitrogen, Carlsbad, CA) for 1 hour at room temperature. Nuclear counterstaining was performed using Vectashield H–1200 mounting medium with DAPI (Vector Laboratories, Burlingame, CA). A Zeiss Axioplan–2 microscope equipped with Axiocam HRc digital camera (Zeiss, Thornwood, NY) was used for imaging.

### The *in vivo* mouse calvarial defect model

To evaluate the *in vivo* calvarial healing capacity of *Axin2* ^*-/-*^ and wild type mice, 7-month old mice underwent calvarial defect procedures as previously described[4, 6]. Briefly, after anesthesia 2-millimeter calvarial defects were created with a trephine drill bit in frontal or parietal bones. Meticulous care was taken in order to protect the underlying dura mater or neighboring cranial sutures.

### Micro-CT analysis

μCT-scanning was performed as previously described[4]. Each mouse was scanned with a CT-phantom, which was used to calibrate each scan. The precise threshold for regenerating calvarial bone was previously determined equivalent to 510 Houndsfield Units. The rest-defect area (not healed defect area) was then determined with the Magic Wand Tool in Photoshop (Adobe Systems, San Jose, CA). Percentage healing was determined by dividing the rest defect area by the mean of the defect size one day postoperatively. Mice were scanned 24 hours post-surgery and at week 2, 4, 6 and 8. For statistical analysis, was used the Mann-Whitney test. A *p-value < 0.05 was considered statistically significant.

### Specimens harvesting and histological staining

Skulls were harvested under a stereomicroscope and fixed in 0.4% of Para-formaldehyde (PFA) overnight at 4°C and decalcified in 19% EDTA. Samples were then dehydrated, paraffin embedded and sectioned onto 5 μm using a microtome. The cell proliferative cell nuclear antigen (PCNA) staining was performed using a PCNA staining kit (#93–1143 Invitrogen, Carlsbad, CA) according to manufacturer’s instruction. To detect apoptotic activity, was used the ApopTag plus peroxidase in situ apoptosis detection kit (#S7101, Millipore, Billerica, MA). Briefly, slides were pretreated with proteinase K for 15 minutes, followed by incubation with 3% hydrogen peroxide at room temperature for 3 minutes to quench the endogenous peroxidase. Then, slides were incubated with the deoxynucleotidal-transferase enzyme (TdT) for 2 hours at room temperature and counterstained with methyl green. All the procedures were conducted in a moisture chamber. For bony tissue assessment Movat’s modified pentachrome staining was performed on coronal sections derived from calvarial defects at week 8 post-surgery. All histological sections were examined under a Zeiss Axioplan microscope equipped with an Axiocam HRc digital camera (Zeiss, Thornwood, NY).

### Statistical analysis

Both the *in vitro* or *in vivo* experiments in this study were designed as either triplicate or repeat two or three times. The data are expressed as mean ± SD of three independent experiments. The error bars in the graphs represent one standard deviation. Statistical differences between the means are examined by Student’s test. A *p value <0.05 was considered statistically significant.

## Results

### *In vitro* analysis of osteogenic potential of *Axin2* ^*-/-*^ FOb and POb

**Fig 1:**
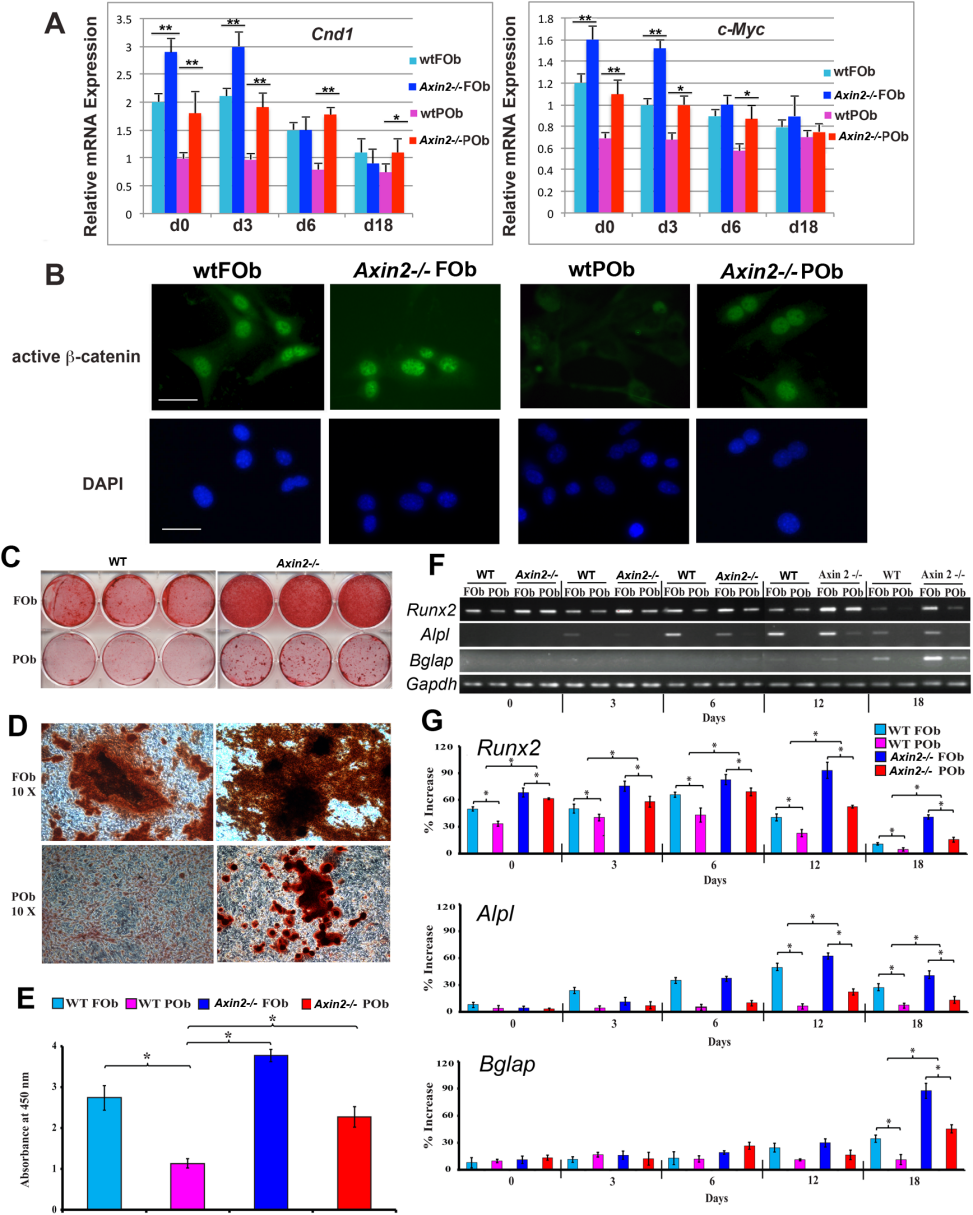
*In vitro* differentiation and osteogenic-related gene expression analysis of *Axin2* ^*-/-*^ and wild type FOb and POb cells. (**A)** quantitative PCR analysis performed on wt and *Axin2* ^*-/-*^FOb and POb showing a significant upregulation of cWnt signaling target genes in both, *Axin2* ^*-/-*^FOb and POb as compared to wt osteoblasts. (**B**) enhanced activation of cWnt signaling in *Axin2* ^*-/-*^FOb and POb is further confirmed by indirect immunofluorescence analysis showing larger number of cells with positive nuclear staining for active β-catenin. Positive nuclear staining is more dramatic in *Axin2* ^*-/-*^POb. Nuclear counterstaining was performed with DAPI (objective magnification 10x). Scale bars = 50μm. (**C**) Cells were cultured with differentiation medium for 18 days. Mineralization of extracellular matrix as assessed by alizarin red staining indicates a more robust mineralization in *Axin2* ^*-/-*^ FOb and POb as compared to corresponding wild type FOb and POb. (**D**) Magnification of alizarin red stained bone nodules. (**E**) Quantification of alizarin red staining as showed above (panel A) confirms enhanced osteogenic capacity of FOb and POb cells derived from *Axin2* ^*-/-*^ mice. (**F**) RT-PCR analysis of osteogenic markers showing significant higher up-regulation of the *Runx2*, *Alpl* and *Bglap* in *Axin2* ^*-/-*^ FOb and POb. (**G**) Histograms representing quantification of each electrophoretic band obtained by Image J program. Each band was normalized to its *Gapdh* content. *P≤ 0.05.

Firstly, enhanced activation of cWnt signaling in *Axin2* ^*-/-*^ FOb and POb was confirmed by significantly up regulation of the target genes of cWnt signaling *Cyclin D1* and *c-Myc*, as well by intense nuclear staining for active β-catenin in *Axin2* ^*-/-*^ POb cells as compared to wild type POb (Fig 1A and 1B). Next, we assessed the osteogenic capacity of FOb and POb isolated from neural crest-derived frontal bone and mesoderm-derived bone of pN21 *Axin2* ^*-/-*^ and wild type mice, respectively. Cells of each group were seeded at density of 1.5 x 10^5^ cells per well in 6 well plates to induce osteogenic differentiation using ODM. After 18 days, the mineralization of extracellular matrix was determined by alizarin red staining and its quantification. As shown in Fig 1C–1E, the extracellular matrix mineralization was more robust in both *Axin2* ^*-/-*^ FOb and POb as compared to wild type FOb and POb cells. Quantification of alizarin red staining indicated a significant increase of mineralization in *Axin2* ^*-/-*^ POb to a level similar to that of wild type FOb, whereas POb mineralized poorly (Fig 1E) as previously reported [6, 8, 9].

A significant increase in mineralization was also observed in *Axin2* ^*-/-*^ FOb when compared to corresponding wild type osteoblasts. The increased osteogenic profile observed in *Axin2* ^*-/-*^ FOb and POb was confirmed at molecular level by the expression of osteogenic markers, such as the early marker *Runx2*, the intermediate alkaline phosphatase (*Alpl*) and late osteocalcin (*Bglap*) markers (Fig 1F and 1G). Interestingly, at day 0 prior differentiation started, we observed significant higher levels of endogenous *Runx2* in *Axin2* ^-/-^ FOb and POb compared to wild type. This observation would suggest the presence of a larger subpopulation of osteoprogenitor cells in *Axin2* ^-/-^ cells. At day 18, marking terminal osteogenic differentiation, a significant higher expression of the late osteogenic marker (osteocalcin) *Bglap* was found in both *Axin2* ^*-/-*^ osteoblasts, with POb expressing level of *Bglap* higher than corresponding wild type FOb. Of note, increased expression of osteogenic markers such as *Runx2* and *Alpl* was detected also in frontal and parietal bone tissues harvested from *Axin2-/-* mice compared to wild type bones (S1 Fig).

Taken together, these results indicate that enhanced activation of cWnt signaling increased not only the osteogenic potential of calvarial osteoblasts but conferred to mesoderm-derived osteoblasts an osteogenic ability similar to that of neural crest-derived osteoblasts.

### Cell proliferation properties of *Axin2* ^*-/-*^ FOb and POb and calvarial bones

**Fig 2:**
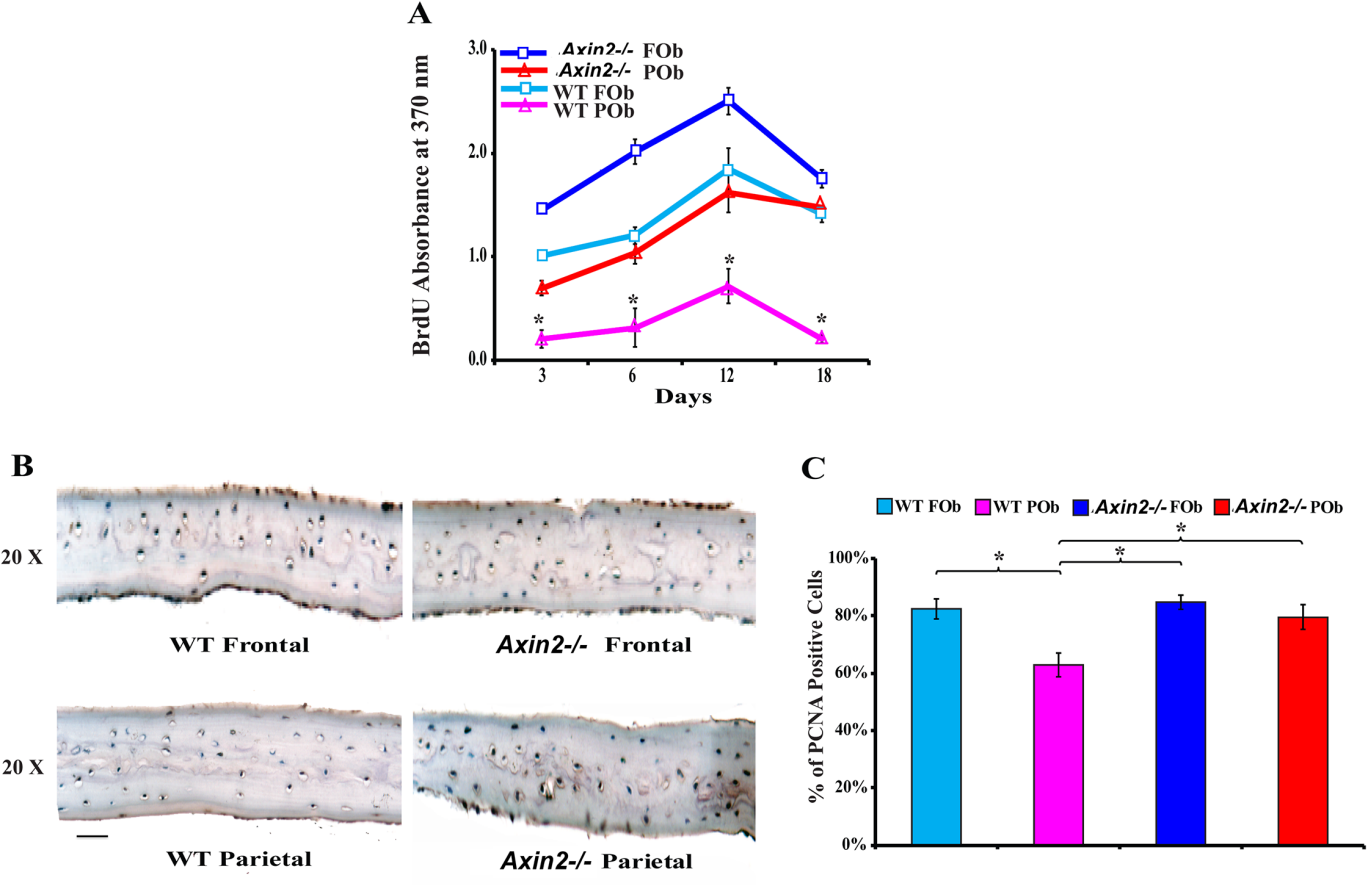
Enhanced proliferative activity of *Axin2* ^*-/-*^ FOb and POb. (**A**) *In vitro* BrdU assay performed on *Axin2* ^*-/-*^ and wild type FOb and POb cells undergoing differentiation reveals increased proliferative activity in *Axin2* ^*-/-*^ cells than corresponding wild type. (**B**) *in vivo* PCNA immunostaining performed on coronal sections derived from frontal and parietal bones of pN21 *Axin2* ^*-/-*^ and wild type mice, also indicates an increase in proliferation of *Axin2* ^*-/-*^ frontal and parietal bones compared wild type. (**C**) Quantification of PCNA staining obtained by calculating the percentage of PCNA positive cells over the total cell number counted at least in five equivalent areas of each bone, indicates the lowest cell proliferation activity in wild type parietal bone, whereas *Axin2* ^*-/-*^ parietal bone displays activity similar to that of wild type frontal bone. Scale bar = 150 μm.

We have previously demonstrated that in addition to greater osteogenic capacity, neural crest-derived FOb proliferate more than mesoderm-derived POb[8]. Herein, we addressed whether enhanced activation of cWnt signaling would impact the proliferative activity of POb making them more like FOb. To this aim, the proliferation rate of either *Axin2* ^*-/-*^ FOb and POb or corresponding wild type osteoblasts, undergoing to osteogenic differentiation, was measured by BrdU incorporation. This assay showed overall more proliferation in *Axin2* ^*-/-*^ FOb and POb, with the latter displaying a proliferation rate similar to that of wild type FOb. Whereas, wild type FOb and POb showed a growth proliferation pattern similar to that reported in earlier studies[8], with FOb proliferating significantly more that POb (Fig 2A). By day 18, when terminal osteogenic differentiation was achieved, the proliferation dramatically decreased in all cells.

The proliferative cell activity was also assessed *in vivo* by immunostaining of the proliferative cellular nuclear antigen (PCNA) performed on coronal sections derived from frontal and parietal bones of *Axin2* ^*-/-*^ and wild type pN21 mice (Fig 2B). Quantification of PCNA positive stained cells calculated as a percentage of the total cells observed in similar areas of each bone, indicated higher percentage of PCNA positive cells in *Axin2* ^*-/-*^ parietal bone than corresponding wild type bone (Fig 2C). Again, as observed *in vitro*, the proliferative state of *Axin2* ^*-/-*^ parietal bone was significantly elevated compared to wild type, with a proliferative rate similar to that of wild type frontal bone. Thus, the *in vivo* proliferative state of *Axin2* ^*-/-*^ frontal and parietal bones mirrored the *in vitro* profile.

### Decreased apoptotic activity in *Axin 2* ^*-/-*^ FOb and POb and calvarial bones

**Fig 3:**
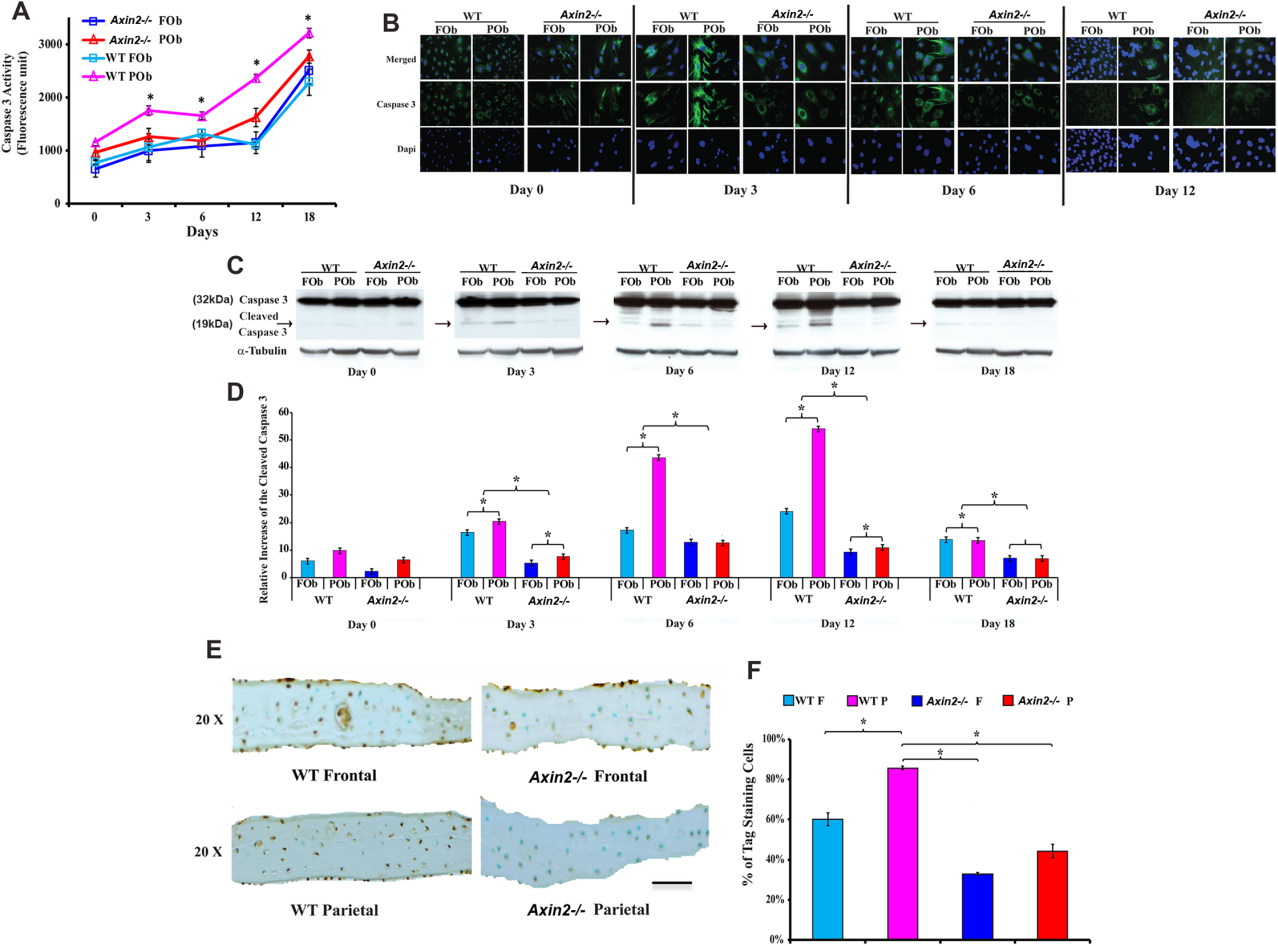
Decreased apoptotic activity in *Axin2* ^*-/-*^ FOb and POb and calvarial bones. (**A**) Apoptotic activity in *Axin2* ^*-/-*^ FOb and POb undergoing to differentiation measured as Caspase 3 fluorescence units. *Axin2* ^*-/-*^ FOb and POb cells show similar level of Caspase 3 activity, and wild type POb the highest level. Apoptosis increased over time during the osteoblast differentiation. (**B**) Caspase 3 indirect immunofluorescence analysis showing intense nuclear and cytoplasmic fluorescence in wild type POb at day 3 and 6 of osteogenic differentiation, whereas only faint cytoplasmic staining is observed in *Axin2* ^*-/-*^ POb. (**C**) Detection of active cleaved Caspase 3 by immunoblotting analysis. A 19 kDa band corresponding to cleaved Caspase 3 is detected in wild type POb starting from day 3, of osteogenic differentiation. (**D**) Histograms representing quantification of each electrophoretic band obtained by Image J program. Each band was normalized to α-tubulin loading control. (**E**) TUNEL staining performed on coronal sections derived from *Axin2* ^-/-^ and wild type parietal and frontal bones harvested from pN21 mice. (**F**) Quantification of the Caspase 3 activity in bones by calculating the percentage of the positive Tag stained cells (brown stain) over the total cells in similar areas of the bone shows the highest apoptotic activity in parietal bone of wild type mice, while apoptotic activity in parietal bone of *Axin2* ^*-/-*^mice is significantly reduced. Scale bar = 150 μm.

Having observed substantial difference in proliferation between *Axin2* ^*-/-*^ and wild type FOb and POb, we next sought to investigate apoptosis in these cells, since the implication of cWnt signaling in apoptotic activity has been reported previously [23]. Therefore, by using an ELISA assay, we measured the Caspase 3 activity in *Axin2* ^*-/-*^ and wild type FOb and POb undergoing osteogenic differentiation. As shown in Fig 3A, a specific pattern characterized overall by increased apoptotic activity during the late time points of osteogenic differentiation, could be observed in all four groups. Interestingly, markedly lower levels of Caspase 3 activity were detected in POb derived from *Axin2* ^*-/-*^ mice as compared to wild type POb cells, thus showing a “FOb-like” profile. Moreover, wild type POb displayed significantly higher Caspase 3 activity than wild type FOb cells as previously shown[9]. A similar profile was also observed by performing immunofluorescence analysis using a specific anti-Caspase 3 antibody. As shown in Fig 3B, Caspase 3 nuclear and cytoplasmic staining was detected in wild type FOb and POb, with more intense staining in POb, while only a faint cytoplasmic staining was observed in *Axin2* ^*-/-*^ osteoblasts. In addition, active cleaved Caspase 3 was visualized at protein level by immunoblotting analysis performed on cell lysates of osteoblasts at different time points of osteogenic differentiation. This analysis revealed higher levels of cleaved Caspase 3 in wild type FOb and POb with the latter having the highest level (Fig 3C and 3D).

Next, to investigate whether a similar apoptotic profile would exist *in vivo*, we performed staining on coronal sections of frontal and parietal bones harvested from *Axin2* ^*-/-*^ and wild type pN21 mice. As revealed by ApopTag (TUNEL) staining, *in vivo* apoptotic activity mirrored the *in vitro* apoptotic profile (Fig 3E and 3F). Both frontal and parietal bones of *Axin2* ^*-/-*^ mice displayed significantly lower apoptotic activity than corresponding wild type bones. Importantly, the level of apoptosis in *Axin2* ^*-/-*^ parietal bone was even lower than in wild type frontal bone, and further decreased in *Axin2* ^*-/-*^ frontal bone. Quantification of ApopTag staining obtained by counting Tag-positive cells (brown staining) and calculated as a percentage of the total cells in equivalent areas of frontal and parietal bony tissues confirmed the observation that apoptotic activity was significantly less in *Axin2* ^*-/-*^ frontal and parietal bones, whereas wild type bones displayed the highest apoptotic activity. Collectively, these data demonstrate that enhanced activation of cWnt signaling impacted significantly apoptosis in calvarial bones of different tissue origin, specifically by decreasing this activity in parietal bone to a level even lower than that observed in wild type frontal bone.

### Assessment of bone regeneration in *Axin2* ^*-/-*^ mice using a calvarial defect healing model

**Fig 4:**
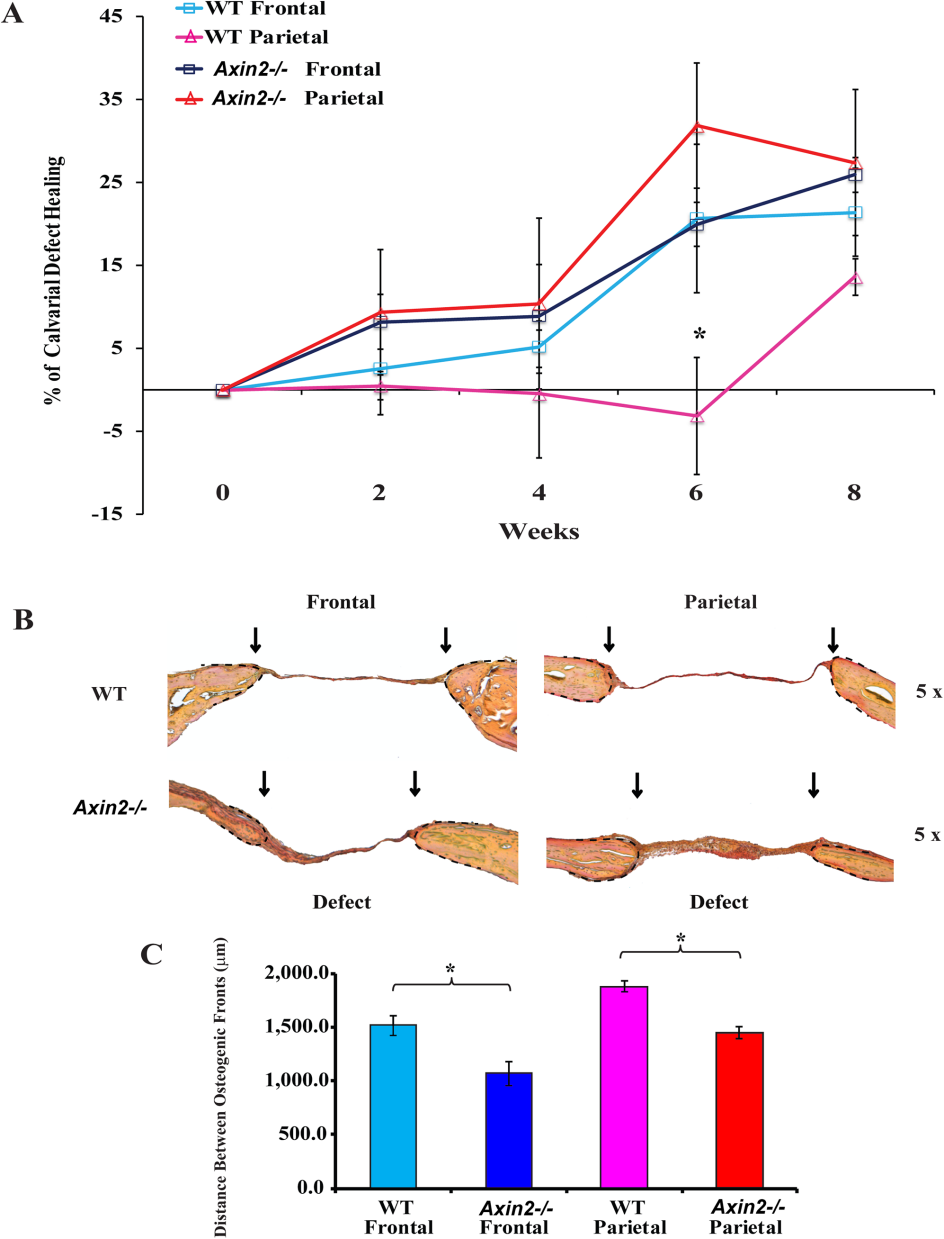
*In vivo* calvarial healing of *Axin2* ^*-/-*^ and wild type frontal and parietal bones. **(A**) Two-millimeter (2mm) defects were created in the frontal and parietal bones of 7 month-old *Axin2* ^*-/-*^ and wild type mice (n = 3). Quantification of defect repair according to microCT-scan results. Statistical analysis was conducted utilizing the Mann-Whitney Test. P-values: *P≤ 0.05. (**B**) Pentachrome staining of coronal sections of skull at post-operative week 8 showing the repair of calvarial bone defects as determined by yellow color. Bone regeneration was higher in *Axin2* ^*-/-*^ frontal and parietal bones as compared to wild type bones. **(C)** Histogram showing the distance between the osteogenic fronts (dashed) and marked by arrows (Objective magnification 5x).

Last step of our investigation was to determine the impact of enhanced cWnt signaling in regeneration of frontal and parietal bones. To this end, 2-mm calvarial defects were created in frontal and parietal bones of 7-month old *Axin2* ^*-/-*^ and wild type mice (n = 3). The healing process was monitored by micro-CT scan at week 0, 2, 4, 6, and 8 post-surgery. A significant difference in healing rate among the groups was observed. As illustrated in Fig 4A, wild type frontal bone defects healed faster than parietal bone, thus confirming previous results[6]. In *Axin2* ^*-/-*^ groups, starting from week 2 post-surgery, we observed faster healing rates, in both frontal and parietal bones, compared to the wild type. At week 6, the healing of *Axin2* ^*-/-*^ parietal defect healing was higher than the *Axin2* ^*-/-*^ frontal defect healing, as determined by defect percent closure.

To assess for bone healing at histological levels, coronal sections of skull with bone defects were stained with pentachrome procedure. Pentachrome staining of skull defects, harvested 8 weeks after surgery, confirmed the superior healing capacity of *Axin2* ^*-/-*^ frontal and parietal bone defects as compared to wild type defects (Fig 4B and 4C). There was only limited bone regeneration in wild type frontal defects and almost none in wild type parietal defects. In contrast, *Axin2* ^*-/-*^ frontal and parietal defects showed more bony tissue regeneration. The most robust tissue regeneration was observed in *Axin2* ^*-/-*^ parietal defects showing marked bone matrix formation.

## Discussion

Several studies have indicated that multiple signaling pathways are involved in the regulation of osteogenesis and bone formation in mouse and human[10, 24]. Our previous reports on calvarial bones of different embryonic tissue origin[25] have shown that integration of multiple and differentially activated signaling pathways regulate osteogenesis and bone tissue repair [4, 6, 8, 9, 17].

Based on previous evidence that cWnt signaling is critical in mediating the greater *in vitro* osteogenic potential of FOb compared to POb [6], in the current study we have validated *in vivo* the importance of this signaling to control the repair of calvarial bones of different embryonic tissue origin[25]. To this end we have chose a transgenic mouse with targeted disruption of *Axin 2* [19], a molecule playing a key role in regulating the stability of β-catenin and therefore, the cell response to Wnt signaling[26–28]. This transgenic mouse is characterized by enhanced activation of cWnt signaling due to the targeted disruption of a negative regulator on cWnt signaling[19]. The enhanced activation was further confirmed by significantly upregulation of the target genes of cWnt signaling *Cyclin D1* and c-*Myc*, as well by intense nuclear staining for active β-catenin *Axin2* ^*-/-*^ POb cells as compared to wild type POb. A similar upregulation was observed also in *Axin2* ^*-/-*^ frontal and parietal bone tissues (data not shown). These results confirmed previous *in vitro* and *in vivo* observations [6, 19].

Using this transgenic model we have investigated the coordinated regulatory role played by cWnt signaling pathway in calvarial osteoblast differentiation and regeneration of bones with different embryonic tissue origin.

Calvarial bone formation is the result of a proper balance between osteoblast recruitment, proliferation, differentiation and apoptosis. Many studies have shown that the cWnt signaling pathway plays an important role in the osteogenesis *in vivo* and *in vitro*, as reviewed earlier. Cell proliferation and apoptosis are among the major roles played by active cWnt signaling [23, 29–31]. Having observed a major osteogenic potential in *Axin2* ^*-/-*^ POb cells as compared to wild type POb, we investigated the degree of proliferation and apoptosis between wild type and *Axin2* ^*-/-*^ POb cells. Our results indicate that the rate of proliferation either in *Axin2* ^*-/-*^ parietal bone or POb was significant higher than in wild type controls. In addition, analysis of apoptotic activity by caspase 3 enzymatic activity and staining revealed a significant decrease of this activity in *Axin2* ^*-/-*^ parietal bone and POb. Our results confirm cWnt signaling as negative regulator of osteoblast apoptosis supporting previous studies[31]. For instance, Almeida and coworkers demonstrated that Wnt proteins prevent apoptosis of both uncommitted osteoblast progenitors and differentiated osteoblasts by β-catenin-dependent signaling[31]. An independent study performed using a knock out mouse model for sFRP–1, an inhibitor of cWnt signaling, reported decreased apoptotic activity paralleling with increased proliferation in calvarial osteoblasts [32, 33].

In contrast, Yu and colleagues in their study using the *Axin2* ^*-/-*^ mouse model did not observe significant differences in apoptosis activity between *Axin2* ^*-/-*^ and wild type osteoblasts *in vitro*[19]. However, they described a significant increase in proliferation activity as well as osteogenic potential in *Axin2* ^*-/-*^ calvarial osteoblasts compared to wild type[19]. In this context, it must be pointed out that the authors used mainly fronto-nasal osteoblasts for their *in vitro* study and the procedure described to isolate the calvarial osteoblasts was quite different from our procedure which requires the removal of pericranium, dura mater and cranial sutures tissues, prior mechanical and enzymatic digestion of frontal and parietal bone. Moreover, in our procedure we discarded the first two enzymatic digestions to ensure for the purity of FOb and POb isolation, and avoid contamination of undifferentiated cells derived either from pericranium or dura mater which could alter and/or mask intrinsic properties of osteoblasts derived from frontal and parietal bones. Therefore, any potential difference between our and their results might reflect the degree of osteoblasts purity. During this study we observed significantly increased proliferation and decreased apoptosis with enhanced osteogenic differentiation and bone repair in *Axin2* ^*-/-*^ mice compared to wild type. The higher cell proliferation and lower apoptotic activities observed at early time points, (before day 12) during *in vitro* differentiation led to an enhanced bone nodules formation and extracellular matrix mineralization. At later stages, cell proliferative activity sharply decreased, while apoptosis either remained elevated or at a steady level. Apoptosis is needed for the bone remodeling process in the late stage as reported earlier [23]. Indeed, higher cell proliferation ability and lower level of apoptosis are key factors in providing pool of progenitor cells in the early stage of osteogenic differentiation. These data, together with the significantly higher level of *Runx2* expression, would suggest an enrichment of osteoprogenitors in *Axin2* ^*-/-*^ osteoblasts compared to wild type cells.

In order to confirm the *in vitro* observation we investigated the bone repair ability of frontal and parietal bones in *Axin2* ^*-/-*^ mice. To this end, we employed a well establish calvarial defect healing model. Using this model, we have previously shown that significant differences in bony tissue repair between the neural crest-derived frontal bone and the paraxial-mesoderm derived parietal bone exist [6]. We chose to create a 2-mm calvarial defect on adult, 7-month old mice rather than in juvenile mice because older animals have less calvarial healing capability [11, 34]. Therefore, adult mice would provide a more “stringent” condition to unveiling differences in bone-skeletal repair between *Axin2* ^*-/-*^ and wild type mice. Additionally, our choice was also encouraged by an interesting observation obtained from an *in vivo* study performed on sFRP knockout mice showing that significant differences in enhanced bone mass and suppression of senile bone loss was detected only after 13 week of life and reached a peak at 38 weeks postnatally [33].

It is remarkable, how the *in vitro* proliferation and apoptosis profile of FOb and POb derived either from *Axin2* ^*-/-*^ or wild type mice was precisely mirrored *in vivo*. As determined by micro-CT analysis performed at different weeks post-surgery, the healing ability of *Axin2* ^*-/-*^ parietal bone was enhanced compared to wild type parietal bone. Importantly, the pattern of *Axin2* ^*-/-*^ parietal bone healing mirrored that of wild type frontal bone. The latter *in vivo* result thus, confirms that enhanced activation of cWnt signaling is at least one of underlying mechanism(s) conferring a enhanced skeletal potential/repair ability to mesoderm-derived calvarial bones, and importantly, making them like a neural crest-derived bone.

## Supporting Information

S1 Fig Increased expression of osteogenic markers in *Axin2* ^*-/-*^ frontal and parietal bones. **(A)** qPCR analysis of *Runx2* and *Alpl* performed on frontal and parietal bone tissues harvested from WT and *Axin2* ^*-/-*^ pN21 mice reveals higher levels of both osteogenic markers in *Axin2* ^*-/-*^ mice as compared to WT controls.
(TIF)
