# Supplementary figures and images for: Enhanced Activation of Canonical Wnt Signaling Confers Mesoderm-Derived Parietal Bone with Similar Osteogenic and Skeletal Healing Capacity to Neural Crest-Derived Frontal Bone
Source: PLoS One. 2015 Oct 2;10(10):e0138059. doi: 10.1371/journal.pone.0138059 (PMC4592195; doi:10.1371/journal.pone.0138059)

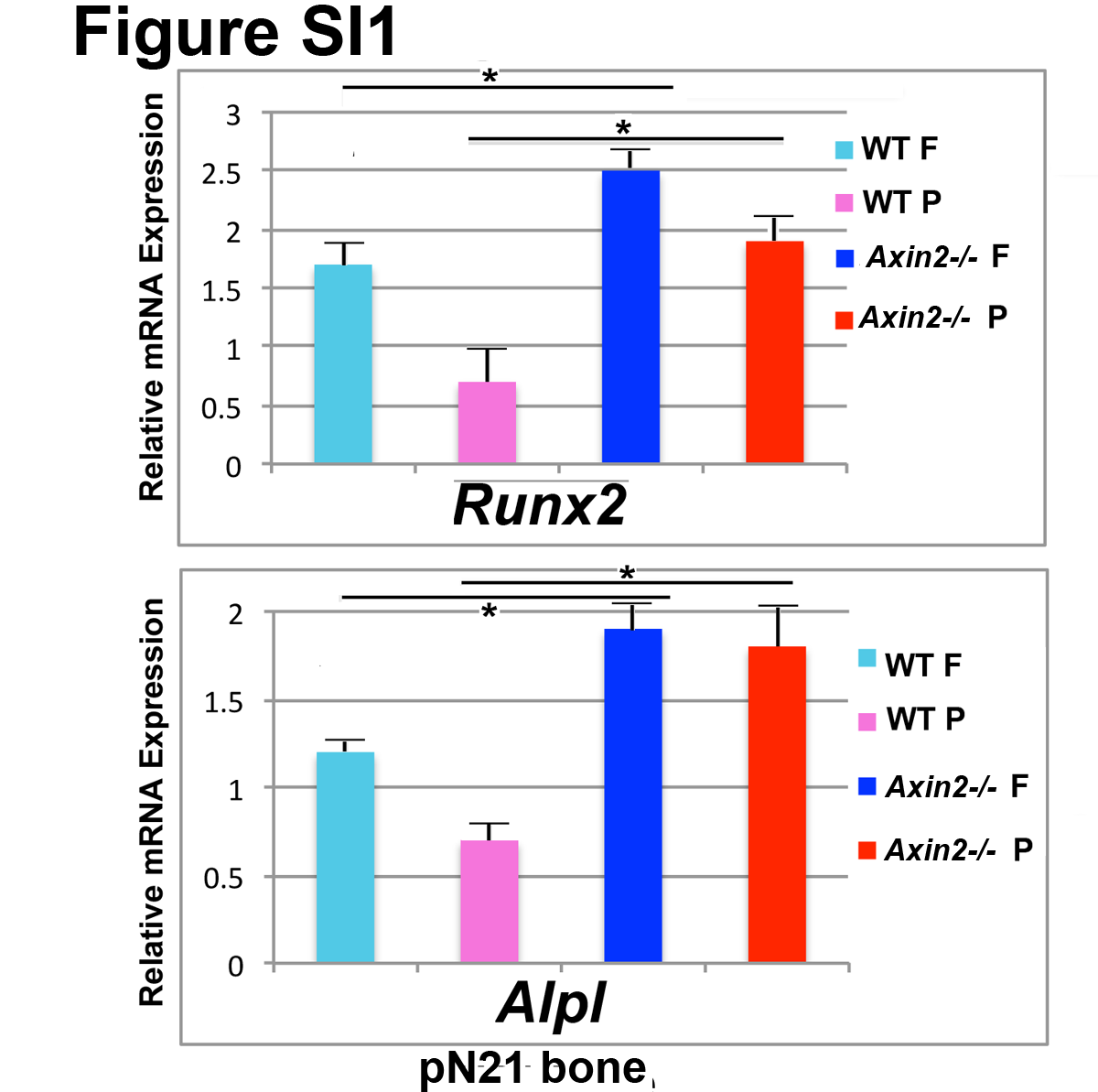

Supplement: S1 Fig — (A) qPCR analysis of Runx2 and Alpl performed on frontal and parietal bone tissues harvested from WT and Axin2 -/- pN21 mice reveals higher levels of both osteogenic markers in Axin2 -/- mice as compared to WT controls. (TIF) [file pone.0138059.s001.tif]
